# An Automated Framework for Large Scale Retrospective Analysis of Ultrasound Images

**DOI:** 10.1109/JTEHM.2019.2952379

**Published:** 2019-11-19

**Authors:** Pradeeba Sridar, Ashnil Kumar, Ann Quinton, Narelle June Kennedy, Ralph Nanan, Jinman Kim

**Affiliations:** 1 School of Computer ScienceThe University of Sydney 4334 SydneyNSW2006Australia; 2 School of Biomedical EngineeringThe University of Sydney 4334 SydneyNSW2006Australia; 3 Sydney Medical School NepeanThe University of Sydney 4334 SydneyNSW2006Australia; 4 School of Health, Medical and Applied SciencesCentral Queensland University 6939 SydneyNSW2000Australia; 5 Charles Perkins CentreThe University of Sydney 4334 SydneyNSW2006Australia

**Keywords:** Automated framework, fetal ultrasound, clinical repository, classification, measurement

## Abstract

Objective: Large scale retrospective analysis of fetal ultrasound (US) data is important in the understanding of the cumulative impact of antenatal factors on offspring’s health outcomes. Although the benefits are evident, there is a paucity of research into such large scale studies as it requires tedious and expensive effort in manual processing of large scale data repositories. This study presents an automated framework to facilitate retrospective analysis of large scale US data repositories. Method: Our framework consists of four modules: (1) an image classifier to distinguish the Brightness (B) -mode images; (2) a fetal image structure identifier to select US images containing user-defined fetal structures of interest (fSOI); (3) a biometry measurement algorithm to measure the fSOIs in the images and, (4) a visual evaluation module to allow clinicians to validate the outcomes. Results: We demonstrated our framework using thalamus as the fSOI from a hospital repository of more than 80,000 patients, consisting of 3,816,967 antenatal US files (DICOM objects). Our framework classified 1,869,105 B-mode images and from which 38,786 thalamus images were identified. We selected a random subset of 1290 US files with 558 B-mode (containing 19 thalamus images and the rest being other US data) and evaluated our framework performance. With the evaluation set, B-mode image classification resulted in accuracy, precision, and recall (APR) of 98.67%, 99.75% and 98.57% respectively. For fSOI identification, APR was 93.12%, 97.76% and 80.78% respectively. Conclusion: We introduced a completely automated approach designed to analyze a large scale data repository to enable retrospective clinical research.

## Graphical abstract

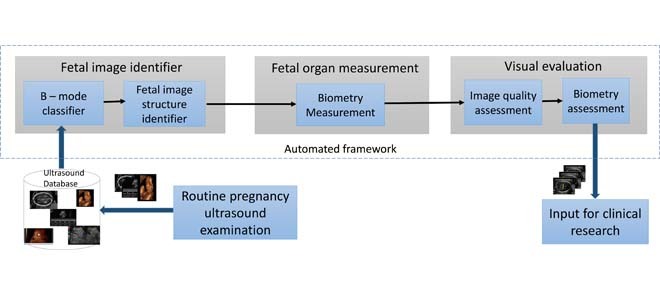

## I. Introduction

Ultrasound (US) screening is the global standard of care for the detection of developmental fetal anomalies during pregnancy [1]. Fetal developmental monitoring is clinically important for risk stratification and interventional purposes. In current clinical practice, monitoring of a limited number of anatomical structures [2]–[4] and their appearance forms a part of the routine assessment of fetal growth, which requires considerable clinical expertise in both US image acquisition and interpretation. However, much more information is embedded in the routinely acquired US images than just the characteristics of the standard set of anatomical structures, and this additional information are currently unused. This information can potentially be used to determine the influence of antenatal factors on fetal development [5], e.g., thalamic size as measured on the US images may be associated with maternal methadone usage and hence may influence neuro-developmental outcomes [6]. This approach requires the understanding the relationship between environmental factors and health outcomes with the fetal growth and anatomical parameters encoded within the US image. These parameters derived from these US images when linked with maternal characteristics (e.g., smoking status, malnutrition, lifestyle, etc.) can help to identify risk factors that contribute to epigenetic modification in the offspring. While hospital imaging repositories are currently used to monitor patient dosimetry, radiographic procedures, and image quality [7], the images within the repositories are not accessible for large scale retrospective analysis as they are not indexed by the content relevant to a specific scientific question (e.g., not indexed by organ or other structures). Hence, tedious and time-consuming manual processes are currently needed to identify and analyze the images that are appropriate to answer specific scientific questions.

**FIGURE 1.**
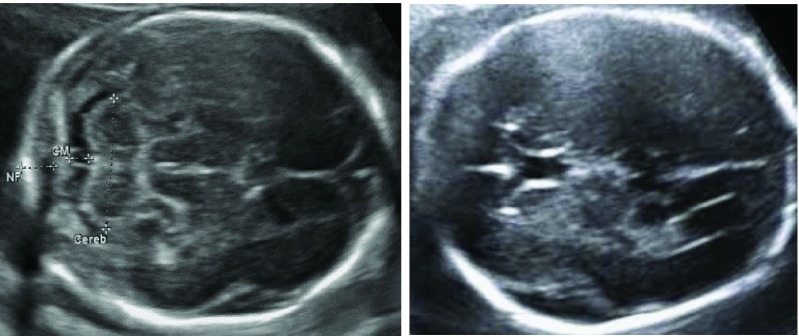
Axial planes of fetal head.

Advances in data-driven approaches such as machine learning (ML) algorithms have made practical breakthroughs and research innovations in medical image analysis. These advances can be effectively used to automate the analyses of US images, as demonstrated in several studies [8]–[11]. However, none of these studies demonstrated the use of a large scale hospital data repository. Properties of large scale hospital data repository that makes automated analysis a complex task are:

• There is a low intra-class variation in fetal images due to the strong visual similarity and structural appearances between the images of same fetal organ [12]. For example, Figure 1 shows two axial planes of the fetal head where the fetal skull and mid line falx is visible in both the images. The low variability in the image appearance arises from different conditions during image acquisition, differences in the machines used for the acquisition, and variability in the skill or the processes of the machine operators [13]. The variation requires automatic methods that are robust in their ability to discriminate the targeted images from the images that have similar structural appearances.
• There is a high degree of variability in images as hospital repositories store data from several patients acquired in many years. For example, there may be variability in image quality due to differences in acquisition at different gestational ages; images of the same structure may also have varying appearances based on the development of the fetus. The automated algorithms must be robust to such variations.
• Hospital repositories contain several types of imaging modalities that fall within the US family, such as Brightness (B) -mode, Motion (M) -mode, pulsed Doppler, Doppler US, US videos, 4D snapshots and dual displays [14]. When the retrospective analysis requires information from only a subset of these modalities, the automatic method must be able to identify the correct modality.
  In this paper, we aim to address these challenges by developing an automated framework to enable retrospective analysis of large scale US data repositories. Our automatic framework leverages state-of-the-art ML and image processing techniques to identify specific images from the hospital repository and extract relevant clinical information from them. Our framework comprises four modules, each of which address a particular image analysis challenge faced in the retrospective analysis of US images: (1) a B-mode image classifier to retrieve the B-mode images (mainly containing anatomical information) from the other US image modalities; (2) a fetal image structure identifier to select the US images that contain specified fetal structure of interest (fSOI); (3) a biometric measurement algorithm to measure the fSOI from the identified images and, (4) a visual evaluation module to allow clinicians to efficiently validate the automated analysis.

Our framework is different from the other automated studies in the following ways:

• It presents an approach to overcomes the challenges in automatically accessing US images in hospital data repositories.
• It has been demonstrated to work on larger datasets (millions of images), while existing automated studies on fetal US images have only been demonstrated on small manually curated datasets.
• It has the ability to extract and analyze specific fSOI from the hospital data repository, demonstrated by our case study of identifying the transcerebellar (TC) plane and measuring the thalamic diameter as fSOI. The TC planes used in this retrospective study were acquired as part of routine fetal examinations and not specifically acquired to visualize the landmarks of the thalamus for research.

## II. Methods and Procedures

### A. Automated Framework

**FIGURE 2.**
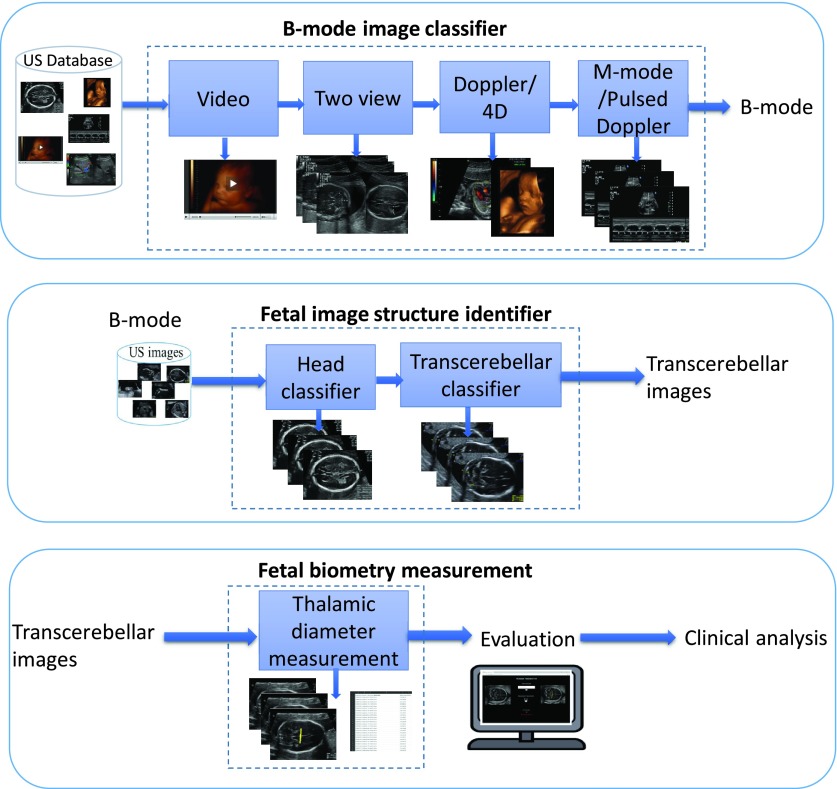
Overview of the proposed framework, modules of the framework are shown inside the dotted blue boxes.

Figure 2 shows the overview of our framework. The modules of B-mode image classifier, fetal image structure identifier, and fetal biometry. They are described in detail within the subsections below.

### B. B-Mode Image Classifier

B-mode is a 2D US image display mode representing the tissues and organ boundaries within the body [15]. In routine fetal scans, it is primarily used for visualization and quantification of fetal anatomical structures. Thus, in a retrospective study, the correct identification of the B-mode images is necessary. We designed our B-mode classifier as a cascade of four filters to retrieve B-mode images from the rest of the US data (Video, dual display, color Doppler, pulsed Doppler and M-mode) using a fast and reliable approach. A separate filter was not designed to remove the 4D snapshots present in the database, as they were subsequently removed by the existing filters in the B-mode image classifier module. The filters were sequential, with the filtered images of one filter becoming the input to the next filter. File mentioned in this classifier refers to a single DICOM object. A US study can have multiple DICOM objects in the form of 2D US images or videos.

#### 1) Video Filter

The video filter was designed to remove cine loop frames from a large US dataset, returning only the 2D images. The filter was operated by leveraging the information stored within the DICOM fields present in the DICOM header of each image [16]. Specifically, the filter checks the value of the DICOM field, “NumberofFrames” (code ‘0028, 0008’) of every US file. If this field was not a part of the DICOM header, then the file was not a video (a multi-frame image). Files without this field and NumberofFrames >1 are categorized as non-video files and passed to the next filter.

#### 2) Dual Display Filter

**FIGURE 3.**
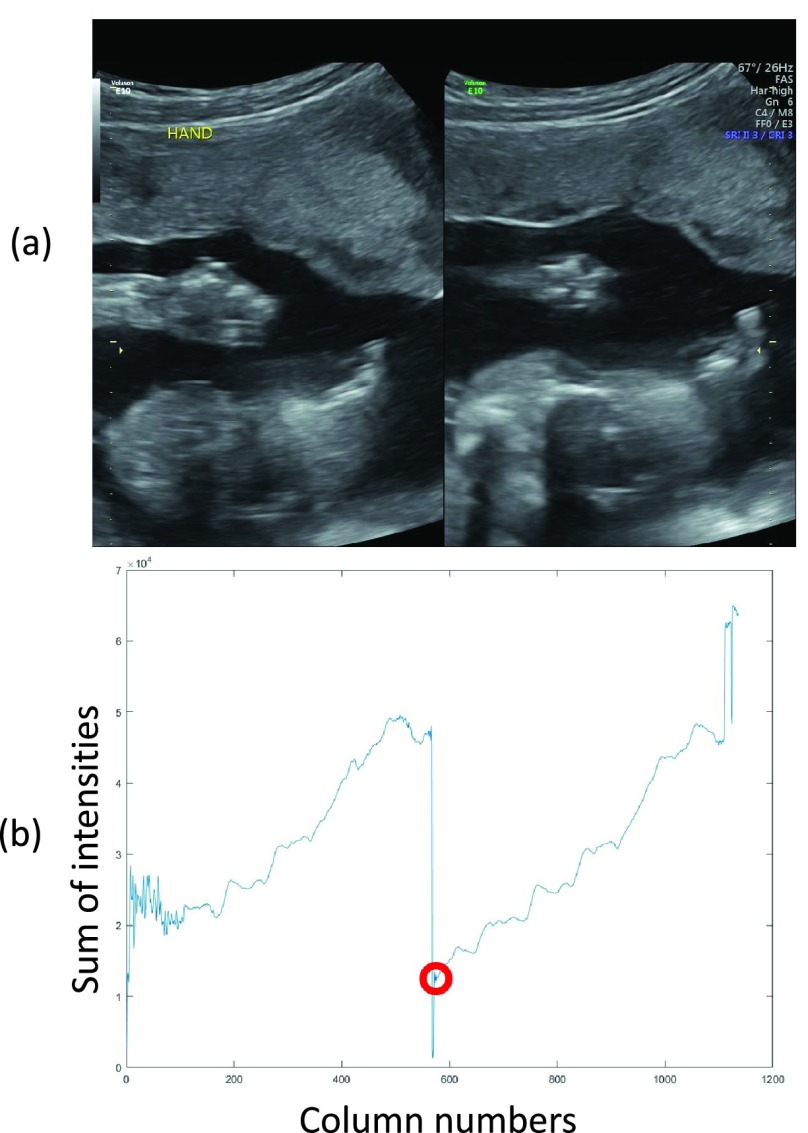
(a) A dual display image. (b) Line profile pattern of the dual display image; the red marker indicates the division between the two US images.

The dual display filter identifies the images captured under a dual display mode, in which two US images were depicted side-by-side within the same frame. An example is shown in Figure 3 (a). The filter was operated by analyzing the intensity profile of the image. The sum of intensities in each image column (x- or horizontal axis) was first computed, and then analyzed to identify the trough in the x coordinate near the middle of the profile. The location of the trough indicates the x-coordinate where the split occurs, shown by the red marker in Figure 3 (b).

#### 3) Doppler Filter

**FIGURE 4.**
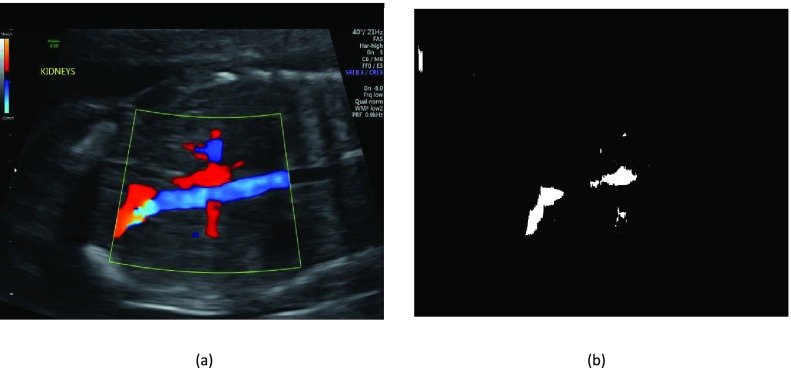
(a) A color Doppler image, and (b) its thresholded mask.

The Doppler filter identifies the Doppler images within the US dataset by thresholding the color information within the image. Doppler images depict the relative shift in the velocity of blood using a color map overlaid on a B-mode image of an organ of interest, allowing clinicians to assess the functionality of the organ [14]. For example in Figure 4 (a), the blue region represents the blood flow away from the transducer while the red region represents the blood flowing toward the transducer. The filter first converts the input US image in the RGB (Red, Green, Blue) color space into the HSV (Hue, Saturation, Value) color space. The HSV image was then thresholded along the V channel with a threshold value of t = 0.1; pixels with values below the threshold were set to zero, while pixels above this value were set to one, as shown in Figure 4 (b). The thresholded mask was identified as a colour Doppler image, if there were non-zero values within the thresholded image.

#### 4) M-Mode and Pulsed Doppler Filter

**FIGURE 5.**
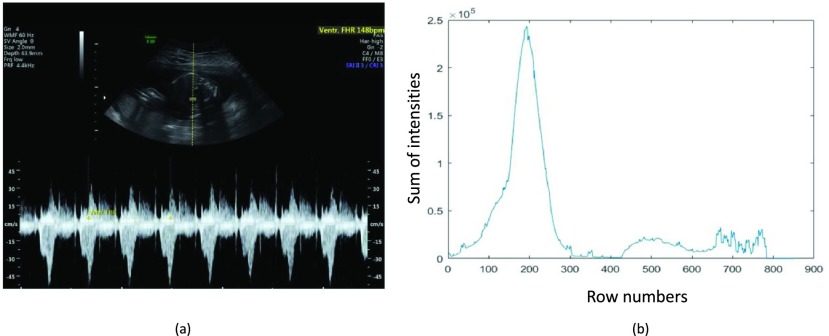
(a) A pulsed Doppler image, (b) Intensity pattern of a pulsed Doppler image.

This filter identifies the images acquired using motion mode imaging (which captures the movement of the fetal heart over time [17]) and pulsed Doppler technique (which was used to examine the velocity flow pattern and/or to perform the signal analysis from a specified region in fSOI [18]). The signal from the fSOI were often displayed below the B-mode image of the structure as shown in Figure 5 (a). The filter was used to analyze the intensity profile of the image and computes the sum of intensities along the vertical (y) axes, as shown in Figure 5 (b). This sum profile was then analyzed to identify peaks in the first and second halves of the signal. If there was a highest peak in the first half with its height (prominence) greater than 50000 and the ratio of the highest peak in the first half to the second half was greater than a threshold of 2.5, then the image was identified as an M-mode/pulsed Doppler image and removed from further processing. The threshold value of 2.5 and the peak prominence value of 50000 was determined through empirical testing. This is the final filter of this module. Hence the images that pass through this filter were the 2D B-mode images to be processed by the other modules.

### C. Fetal Structure Identifier

A cross-sectional analysis of each fetal organ is required to determine their development. In current clinical practice, this assessment occurs by acquiring US images of the fetal organs in utero and then measuring the growth of different anatomical structures at different locations within the fetal anatomy. As the presentation of the fetus can change during image acquisition, the reliability of the measurements obtained from the images largely depends on the correct identification of different structures. Thus, it is necessary to classify the US images into different anatomical viewing planes prior to the analysis, since different anatomical structures appear on different planes.

**FIGURE 6.**
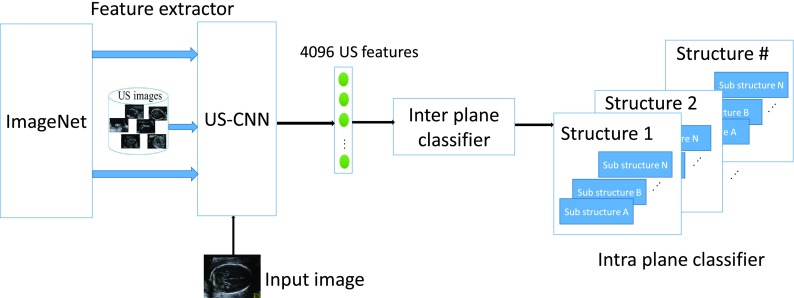
Fetal image classifier model.

The purpose of this module was to classify optimal imaging planes based on the underlying fetal organs and their specific view/planes. As the images could depict different organs and each organ could have multiple views on different planes, we developed a hierarchical classification framework with a feature extractor and a two stage classifier to effectively categorize the fetal images based on organs (inter-plane classification) and the views of an organ (intra-plane classification). The overall framework of the hierarchical fetal image classifier model is shown in Figure 6.

#### 1) Feature Extractor

We used a convolutional neural network (CNN) as a feature extractor to obtain numerical descriptors from US images. A transfer learning approach [19], [20] was chosen to build our network, as the availability of the labelled US fetal images were limited. Fine-tuning is a concept in transfer learning, where a base network learnt from the existing huge datasets (e.g., ImageNet) is retrained on a small dataset (in our case US images) for a target task. We adapted the AlexNet CNN model trained on ImageNet data [21] for US images (US CNN). The last fully connected layer (intended for the 1000 classes of the ImageNet dataset) of the AlexNet architecture was replaced by a new fully connected layer for the 14 different fetal structures in our dataset (Abdomen, Arm, Blood vessels, Cord insertion, Face, Femur and Humerus, Foot, Genitals, Head, Heart, Kidney, Leg, Spine, Hand). The training used the cross entropy loss; the weights of the entire network of the fine-tuned model were iteratively updated using backpropagation with stochastic gradient descent. We used layer specific learning rates to speed up the training process: $10^{-3}$ for our new fully connected layer and $10^{-4}$ for the rest of the network [22]. As the last layer features correspond to more specific features of the US images (target task) than the initial layer features which corresponds to generic image features, we used a higher learning rate for the last layer to enable faster learning of US specific weights for the 14 different fetal classes.

We increased the robustness of the CNN to data variation by augmenting the training dataset. Data augmentation [21] is the process of enlarging the number of the images (data) by transforming the original images and this was performed to reduce the over-fitting during CNN training. Another motivation for using the augmentation was the relatively small set of labeled images in US compared to the other image datasets. The augmentation process generated six variations of the image (original crop c with additional 5 cropping from all the corners and center of c), thereby increasing the number of training samples. The inclusion of such variations of US images in training the CNNs can potentially increase its generalization abilities with new images. We obtained 18654 ($= 3109\times6$ variations) training images after data augmentation. Ninety percent of the augmented training dataset was used for training and 10% for validation. For classification, the images were resized to $227\times227$ and the aspect ratio was preserved during all resize operations by the normalization procedures. Such a resizing operation is standard in transfer learning and fine tuning using CNN’s, as it is a means to transform the new domain (US) to use the input size of the original pre-trained domain [21]. After fine-tuning, the US images and its corresponding crops were represented by a 4096 dimensional feature vector extracted from the last fully connected layer of the US CNN model. Training and testing image features for all the classifiers used in this framework were extracted from the US CNN.

#### 2) Inter Plane Classifier

The B-mode images were fed to the inter-plane classifier to extract the fetal head images. An inter-plane classifier was built by training a multi-class SVM using the features extracted from the fetal US images. We used a linear kernel SVM [23], as it was less prone to over-fitting while non-linear kernel methods requires careful selection of regularization parameters. The training dataset consist of 14 major fetal structures and the class distribution (number of images) is shown in Table 1.

**TABLE 1:** Class Distribution of Training Images of Inter Plane Classification

Fetal Structures	Number of images
Abdomen	106
Arm	174
Blood vessels	389
Cord insertion	127
Face	321
Femur & Humerus	187
Foot	201
Genitals	52
Head	352
Heart	195
Kidney	114
Leg	158
Spine	578
Hand	155

The number of images were not the same through the different classes as it was possible for one organ to be present in multiple views or images. The test set was held out from the training set to avoid bias or overfitting to the test data. At the time of development, this classifier was built as a general framework to classify 14 different fetal US image planes [24]. In this framework, this module was used to classify fetal head images. Readers are encouraged to read our previous work [24] to know further details on the evaluation of inter-plane classifier.

#### 3) Intra-Plane Classifier

Since different planes of any single organ exhibit a high degree of intra-plane similarity in fetal US images, we developed a framework to automatically identify the different planes of any single fetal organ. We overcame the challenge of quantifying the subtle differences in the US images of the same organ by fine-tuning a CNN to derive an US specific feature extractor that was capable of generating image features that were tuned to distinguish US images. We achieved intra plane classification using an ensemble of classifiers to classify the image planes acquired from each organ as shown in Figure 6. In this framework, the intra plane classifier was demonstrated to classify TC plane from the other three axial planes (TC, Transventricular (TV) and Transthalamic (TT)) of the fetal US head. The SVM training was performed using the features extracted from TC images (n = 129) and non-TC images (n = 131; this includes the other axial planes of fetal head) with a linear kernel. Readers are encouraged to read our previous study [12] to know further details on the evaluation of intra-plane classifier.

### D. Fetal Biometry Measurement

Fetal biometry refers to the measurement of the fetal anatomical segments by US [25]. It is important to measure the fetal biometry, as it is used to access the well-being and the growth of the fetus. To measure the fetal biometry, the images obtained from the classifier were next fed to an automated measurement algorithm.

The thalamus cannot be segmented directly due to the lack of well-defined boundaries. We solved this problem by defining a novel algorithm based on statistical shape models (SSMs). We introduced a “guitar” structure that represents the negative space surrounding the thalamus regions. The guitar acts as a landmark for deriving the widest points of the thalamus even when its boundaries are not identifiable. Our method was capable of automatically estimating the thalamus diameter, with the measurement accuracy on par with clinical assessment which has been evaluated in our previous study [26].

**FIGURE 7.**
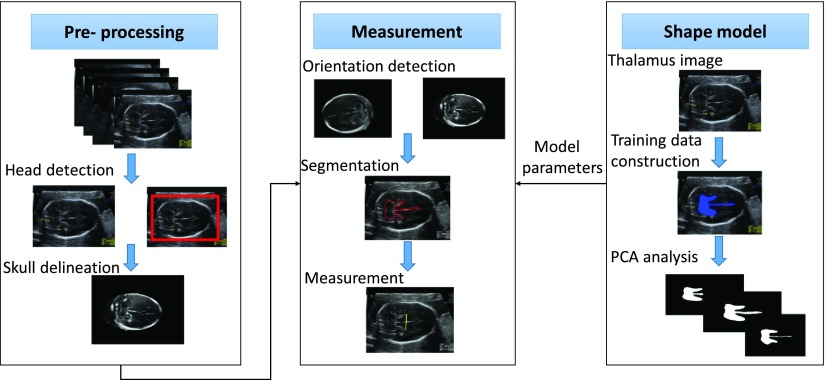
The overall process of the thalamic diameter measurement.

The automated measurement algorithm was built to overcome the inherent limitations of the US image modality: non-uniform density; missing boundaries; and strong speckle noise. The overall process of the measurement algorithm is shown in Figure 7. The TC images were de-noised using a non-linear diffusion filtering algorithm [27], to remove noise and speckle without removing the diagnostic information. As we were interested in the inner contents of the fetal skull, we removed the region outside the fetal skull. It was achieved by delineating the head contour using iterative randomized Hough transform [28] and morphological image processing. Further, as the orientation of the fetal brain can vary based on the position of the fetus and the image acquisition angle, a thalamus specific orientation classifier [26] was developed to detect the orientation of the fetal brain. A novel guitar structure was created from the negative space surrounding the thalamus regions to detect the extremities of the thalamus which otherwise cannot be segmented directly. We augmented a generalized level-set framework with a shape prior and constraints derived from SSM [29] of the guitars from 100 TC images and modified an energy functional [30] with the incorporation of additional shape energy to facilitate guitar segmentation. Line profile analysis method was used for detecting the extremities of the thalamus boundaries. This is the first study to perform computerized measurement of the fetal thalamus diameter. Please refer to our previous work [26] for comparative evaluation of the automated measurements with the manual measurements.

### E. Experimental Setup and Results

#### 1) Implementation Details

All the algorithms were implemented using MATLAB (Mathworks, Natick, Massachusetts, USA). We used a 64 bit PC running Windows 7 Professional as a hardware for processing our framework on a large US dataset. To train the CNN, we used a 12GB NVIDIA Titan X GPU as a hardware. MatConvNet framework was used to train the CNNs.

#### 2) Clinical Dataset

‘eMaternity’ is the fetal dataset hosted by the Department of Obstetrics at Nepean Hospital, Australia. It contains medical records for the fetuses born in the Nepean birth unit between January 2007 and March 2018 inclusive. The women who had undergone their fetal anomaly scans (FAS) between the gestational ages 18 and 22 weeks were included in the cohort study. The images were acquired using 5 different US manufacturers by several sonographers.

Images used for training and testing were obtained using a standard clinical protocol in the routine pregnancy examinations. Experienced sonographers followed the protocol defined by the International Society of Ultrasound in Obstetrics & Gynecology Education Committee [31] for imaging fetal structures during 18–22 week morphology scan. There were no exclusion of images. The machine settings, gain/time gain compensation (TGC), dynamic range and transmit frequency varied according to patient body mass index (BMI) and operator preference. Acoustic output was set at 95%. The zoom scale of each image was adjusted by the sonographers preference based on the maternal BMI and the position of the fetus to ensure the fetal organs were visualized. The zoom level varies markedly and so the association between the BMI and zoom level cannot be assessed. All the US scans were performed within the pelvic-abdominal partition. The US data (2D images and videos) were saved in DICOM format. The ground truth for the training and test images in the development of this framework were provided by two expert sonographer.

#### 3) Evaluation Process

The performance of classifiers was measured using the accuracy ((TP + TN)/(TP + TN + FP + FN)), precision (TP/(TP + FP)), and recall (TP/(TP + FN)), where TP is true positive, TN is true negative, FP is false positive and FN is false negative.

**FIGURE 8.**
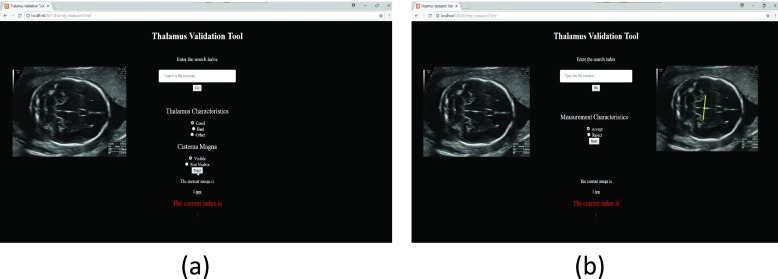
Evaluation viewer: (a) to assess the image quality, (b) to access the automated measurement.

To test the robustness of the developed framework, we used all the data collected in a randomly selected month. This dataset consisted of 1290 US data with 558 B-mode images and 19 TC images. An evaluation viewer was built using web-based tools to ease the process of manual evaluation of the automated outcomes. It has two web pages to assess the quality of the automatically classified images and to assess the automated measurements of fSOI. Sample outcomes representing the visual assessment on a small set of 10 images randomly selected from the test set are shown in Table 3. The table consists of the user assessment of the visual characteristics of the thalamus, cisterna magna and the automated thalamus measurements in the test set. Figure 8 shows the snapshot of the evaluation viewer.

**TABLE 2:** Performance Metrics of the Framework

Stage	Accuracy (%)	Precision (%)	Recall (%)
B-mode image classifier			
Dual display filter	98.36	99.77	98.08
Doppler filter	99.22	100	98.78
M-mode/pulsed Doppler filter	98.45	99.5	98.86
Mean	98.67	99.75	98.57
Fetal image structure identifier			
Inter-plane classifier (Head)	98.20	95.52	90.14
Intra-plane classifier (TC)	88.05	100	71.42
Mean	93.12	97.76	80.78
Mean (framework)	96.45	98.95	91.45

**TABLE 3:** Sample Outcomes of Visual Assessment

	Visibility		Measurement	
File number	TC	CM	Thalamus (cms)	Confirmation
1	Good	Visible	1.69	Accept
2	Good	Visible	2.54	Reject
3	Good	Visible	1.57	Accept
4	Other plane	Not visible	Not available (NA)	NA
5	Good	Visible	2.03	Accept
6	Bad	Visible	NA	NA
7	Good	Visible	1.83	Reject
8	Bad	Visible	NA	NA
9	Bad	Visible	NA	NA
10	Good	Visible	1.43	Accept

### F. Results

#### 1) Quantitative Results of the Automated Framework

We accessed 3,816,967 US files acquired from FAS and identified 1,869,105 B-mode US images and 38,786 fSOI (TC images). The performance metrics of retrieving B-mode images and fSOI on the evaluation dataset is summarized in Table 2.

#### 2) Visual Assessment

**FIGURE 9.**
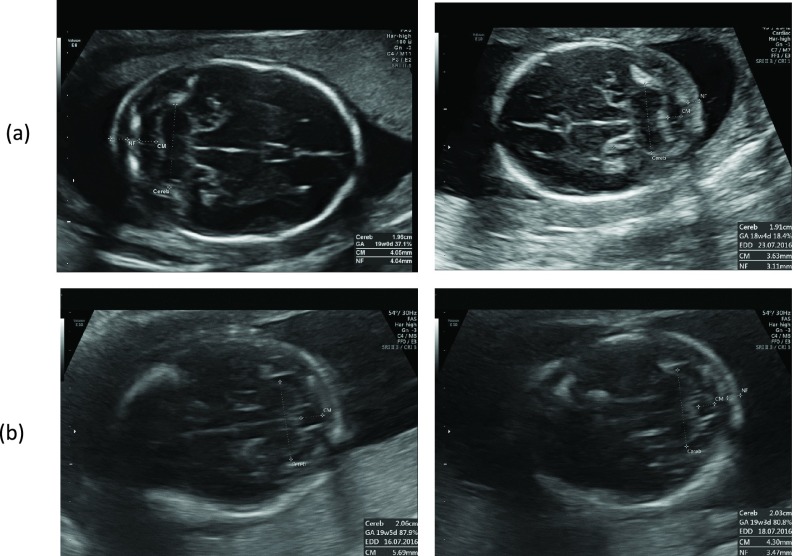
Rows: Image samples of (a) high and (b) low quality images.

**FIGURE 10.**
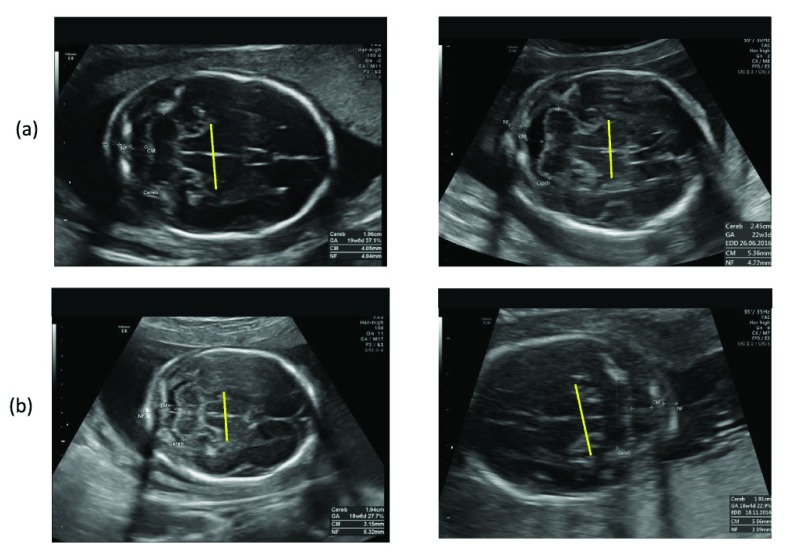
Rows: Image samples of (a) acceptable and (b) non-acceptable measurements.

As the correctness of the measurement depends on the acquired US image plane, the quality of the image planes and measurements were visually assessed by two expert sonographers. The two sonographers involved in the visual assessment had 30 and 25 years of experiences in fetal ultrasonography. Two visual assessments were conducted, independently. Firstly, the image quality assessment was performed adhering to the criteria for the assessment based on the visual characteristics of the fSOI. Secondly, confirmation of the automated measurements was done. The two assessments are shown in Figure 8 using our evaluation viewer. The percentage agreement was 94.73% and the inter-reader kappa values was 0.87 on the test data. These results show an almost perfect agreement between the two sonographers in the visual assessment of the fSOI. Figure 9 shows examples of high quality and low quality TC images. Figure 10 shows examples of automated thalamus measurements on the derived high quality images.

## III. Discussion

Our framework was designed to enable analysis of large scale US data repositories and we demonstrated its utility to retrieve TC images for retrospective analysis. This framework integrates multiple individual modules that we have developed through our prior research [12], [13], [24], [26].

In the fetal US imaging protocol followed by our clinical partners, a routine fetal US study comprises of approximately 50% of B-mode images. Therefore, we deduced that from 3.8 Million US files, we have approximately 1.8 Million B-mode images. The framework produced accurate classification results for the TC plane retrieval with mean accuracy, precision and recall of 96.45%, 98.95% and 91.45%, which is promising as the experimental analysis used routinely acquired images instead of using cleaned images specifically acquired for research. It is be noted that the framework retrieved all the TC planes in the US data repository and did not consider its image quality for classification. Figure 9 shows examples of high and low quality TC images quantified by two expert sonographers based on the visualization of the thalamus and US image attributes such as blur, motion and low contrast ratio.

Figure 10 (b) shows the images with offsets in the measurement, because it was difficult to visualize the landmarks of thalamus. Also, offsets in the measurement can occur in images where the fetal skull is not completely visualized (as shown in the right image in Figure 10 (b)). This is because the fetal skull is an essential anatomical landmark in the TC plane and it was used by the thalamus measurement algorithm for registration. Incomplete imaging of fetal skull occurs when there is a fetal motion or the image is over zoomed during image acquisition.

In this framework, B-mode classifier used a combination of standard established techniques. However, the primary motivation of this study was on the translational feasibility of the framework. While the modules have been published elsewhere, we suggest that the significance of this study is in the end-to-end translation of a pipeline that was used in a clinical environment to analyze huge hospital US database. Further, these published modules had to be optimized to fit the framework and it was evaluated on a large clinical hospital database comprising of complex and diverse data from multiple scanners, parameter settings, operators, etc. From our extensive literature search, this study is the first to introduce a practical automated framework for US data analysis.

In our previous study [12], intra-class classifier had an accuracy of 94.97%. In this study, the accuracy was reduced to 88.05%. We attribute this difference primarily to the more complex and diverse test set used in this study. In the previous study, all test cases were from the same acquisition parameters, while in this study, the test set was representative of the variability of data from different scanners, settings and operators. We note that the precision was on par with previously published results, indicating that there were no false positives. We note that our framework prioritizes precision over recall as it is more important to measure the correct fSOI than to analyze images that may not contain the fSOI.

The 19 TC images in the test set were not pre-selected. Although small when compared to the size of the actual dataset, this set was from all the data collected in a randomly selected month, and we suggest that it is representative of TC measurements. The ground truth for these files was provided manually by the sonographers.

Our framework does not require a special hardware. Once the deep learning modules were built, the framework was efficient in processing the entire database of 3.8 Million files. The average processing time for a single image was 0.13 s and the total processing time in this framework was approximated to 496075.71 s (ie. 6 days).

The future work will include investigations in automatic quantification of US image quality by learning variability’s in US imaging attributes. The framework has generalized abilities which makes it flexible to be optimized to classify and measure any other fSOI from the US data repository.

Currently, this framework is used by sonographers and clinical researchers at the Nepean hospital to access and acquire a large number of fSOI from a hospital data repository. This supports large scale data analysis in clinical studies, which is essential to include potential variability in the control groups. Also, this framework facilitates sonographers by reducing their effort spent in annotating and labeling of stored US images.

## IV. Conclusion

Our framework enabled large scale clinical research that required to access fSOI from a hospital US data repository. We suggest that such capability can facilitate new discoveries e.g., to develop normal range charts or to access structural defects from a large population patient database.

## Ethics and Consent Statements

The Human Research Ethics Committee (HREC) of the Nepean Blue Mountains Local Health District (NBMLHD) has waived informed consent for the use of de-identified images in this retrospective study (HREC reference number: study 07/002).
